# NK Cell Anti-Tumor Surveillance in a Myeloid Cell-Shaped Environment

**DOI:** 10.3389/fimmu.2021.787116

**Published:** 2021-12-17

**Authors:** Eleonora Russo, Mattia Laffranchi, Luana Tomaipitinca, Annalisa Del Prete, Angela Santoni, Silvano Sozzani, Giovanni Bernardini

**Affiliations:** ^1^ Department of Molecular Medicine, Laboratory Affiliated to Istituto Pasteur Italia - Fondazione Cenci Bolognetti, Sapienza University of Rome, Rome, Italy; ^2^ Department of Molecular and Translational Medicine, University of Brescia, Brescia, Italy; ^3^ Humanitas Clinical and Research Center, Istituto di Ricovero e Cura a Carattere Scientifico (IRCCS), Rozzano, Italy; ^4^ Neuromed, Istituto di Ricovero e Cura a Carattere Scientifico (IRCCS), Pozzilli, Italy

**Keywords:** NK cells, macrophages, dendritic cells, chemokine receptors, tumor microenvironment, immunotherapy

## Abstract

NK cells are innate lymphoid cells endowed with cytotoxic capacity that play key roles in the immune surveillance of tumors. Increasing evidence indicates that NK cell anti-tumor response is shaped by bidirectional interactions with myeloid cell subsets such as dendritic cells (DCs) and macrophages. DC-NK cell crosstalk in the tumor microenvironment (TME) strongly impacts on the overall NK cell anti-tumor response as DCs can affect NK cell survival and optimal activation while, in turn, NK cells can stimulate DCs survival, maturation and tumor infiltration through the release of soluble factors. Similarly, macrophages can either shape NK cell differentiation and function by expressing activating receptor ligands and/or cytokines, or they can contribute to the establishment of an immune-suppressive microenvironment through the expression and secretion of molecules that ultimately lead to NK cell inhibition. Consequently, the exploitation of NK cell interaction with DCs or macrophages in the tumor context may result in an improvement of efficacy of immunotherapeutic approaches.

## Introduction

Natural killer (NK) cells are innate lymphoid cells that play a fundamental role in host resistance against cancer thanks to their capacity to directly kill tumor cells and to regulate adaptive tumor immunity.

NK cell activation is regulated by the balance of a set of activating and inhibitory receptors. The major activating NK cell receptors include the natural cytotoxicity receptors (NCR) NKp30, NKp46, and NKp44, as well as the NK group 2 member D (NKG2D), generally engaged by ligands commonly up-regulated on tumor or infected cells as result of cell stress (1–3). Other receptors, including DNAX accessory molecule 1 (DNAM1) and 2B4 act as co-receptors in NK cell activation (4, 5).

In normal conditions, cell surface inhibitory receptors recognize MHC Class I (MHC-I) molecules expressed by healthy cells and inhibit NK cell function as a mechanism of self-tolerance (5, 6). When cells undergo MHC-I downregulation because of infection or transformation, NK cell activation threshold is reduced and activating receptor signaling can be unleashed to promote NK cell cytotoxicity against such cells.

Human blood and tissue NK cells are identified as CD3^-^CD56^+^ (the latter also named neuronal cell adhesion molecule - NCAM) cells and represent a heterogeneous lymphoid population with distinct phenotypic, functional and developmental features. The two most characterized NK cell subsets are an immature CD56^bright^CD16^-^ and a more mature CD56^dim^CD16^+^ population (7). CD56^bright^ cells display a prompter IFNγ response following cytokine stimulation, while CD56^dim^ cells have higher cytotoxic capacity associated with higher levels of granzymes and perforin stored in granules. Tissue trafficking capacity of the two subsets are also distinctively regulated by differential expression of chemotactic receptors: CD56^bright^ preferentially express CCR2, CCR5 and CXCR3 and uniquely express CCR7, while CD56^dim^ have the almost exclusive expression of CXCR1, CX_3_CR1, S1P_5_ and CMKLR1 (8).

The myeloid compartment includes different cell subtypes, such as macrophages and dendritic cells (DC), characterized by high degree of plasticity and heterogeneity, and play important functions in both homeostatic and pathological conditions (9). Macrophages are phagocytes with a key role in microbial clearance and tissue repair whose phenotype strictly depends on their developmental origin, tissue of residence and the environmental stimuli they are exposed to (10). DCs are the most potent antigen-presenting cells (APC) that play an essential role in T cell response and tolerance. DCs are functionally shaped by the integration of tissue environmental signals (11–13). In humans and mice, DCs have been classified as conventional DCs (both cDC1s and cDC2s), plasmacytoid DCs (pDCs), monocyte-derived DCs (moDCs) and Langerhans cells, according to their ontogeny, phenotype, tissue distribution and molecular signature (14). Furthermore, additional subsets with specific roles have been described such as DC3, a pro-inflammatory IRF8-independent subset (15).

## NK Cells, DCs and Macrophages in Cancer

The tumor immune contexture, which defines the amounts and types of tumor-infiltrating leukocytes, has been robustly associated with specific clinical outcomes in patients. The relevance of NK cells in the control of tumor growth and inhibition of metastasis is supported by studies in experimental mouse tumor models and in cancer patients. Indeed, depletion of NK cells in mice bearing transplantation- or carcinogen-induced tumors leads to enhanced tumor aggressiveness and spreading (16, 17). Consistently, also in humans NK cell tumor infiltration is associated with positive prognosis in some solid tumors (18, 19). On the other hand, low NK cell numbers and function are associated with impaired tumor rejection (20), and anti-tumor activity of circulating NK cells was reported to gradually decrease along with progression of certain tumors (21–24). Finally, it is well established that NK cell-based activity can be exploited to treat some cancers, and this is supported by studies of hematopoietic stem cell transplantation (HSCT) starting from the groundbreaking observation by Velardi and colleagues indicating efficacy of haploidentical NK cells transplantation against myeloid leukemias (25). Although NK cell alloreactivity plays a major role in this effect, the efficacy of the treatment is also related to the observation that NK cells are the lymphocytes that first reconstitute upon HSCT (25, 26).

Different DC subsets can localize in or be recruited to tumors where they acquire and process tumor-associated antigens and can thus initiate antigen-specific immunity or tolerance (27).

Tumor-associated monocyte-derived and resident macrophages (TAM) were shown to play a role in the regulation of tumor growth and can support tumor progression by different means, from stimulation of angiogenesis and tumor invasion to suppression of adaptive immune responses (28–31). Roughly, it is possible to distinguish between M1- and M2-polarized macrophages; the first subset arises in response to Toll-like Receptor (TLR) ligands and interferons (IFN) and exerts an anti-tumor function sustained by the potential secretion of pro-inflammatory cytokines, Th1-recruiting chemokines CXCL9 and CXCL10 and the increased expression of MHC-II and costimulatory molecules. On the contrary, M2 macrophages expand in response to IL-4, IL-13, TGF-β and glucocorticoids and exhibit a pro-tumoral phenotype as they are more phagocytic, express higher levels of mannose and galactose receptors and have a highly active arginase 1 (Arg1) pathway (32–35). Nevertheless, this is an over-simplification due to the variety of phenotypes displayed by these myeloid cells that do not often fit the M1/M2 dichotomous phenotypes. Recent single-cell transcriptomic studies show accumulation of several mononuclear phagocyte (MNP) populations in human tumors, some of which with newly described phenotypes related to prevalent pro-tumor activity (36–39). Zang and co-workers identified C1QC^+^ and SPP1^+^ TAM accumulation in colorectal cancer, two populations that differ in phenotypic features related to prevalent pro-angiogenic and immunosuppressive function, respectively (12). By integrating human monocytes and macrophages (MoMac) single-cell RNA obtained from 41 datasets (the MoMac-VERSE), Mulder et al. found that several cancer types display expansion of conserved TAM populations, including IL4I1^+^ PD-L1^+^ IDO1^+^ and TREM2^+^ that expressed mostly M1 and M2 relate genes, respectively (38).

Also location of TAMs in the tumor microenvironment (TME) has been correlated with specific functions, with pathogenic macrophages accumulating at the invasive margin in colorectal cancer, and to negatively correlate with T and NK cell infiltration in several cancer types (36, 38, 39).

DCs, macrophages as well as a heterogenous pool of cells, generally named myeloid-derived suppressor cells (MDSCs), received growing attention in the last decades due to their clear role in determining the polarization of the anti-tumor immune response (40–42).

### The Interaction Between NK Cells and Myeloid Cells

The bi-directional interaction between NK cells and DCs was first described twenty years ago and shown to impact activation, maturation and function of both cell types. Indeed, the crosstalk between immature DCs and NK cells may result in either DC maturation or cell death in regard to the relative DC/NK cell density and DC maturation stage (43, 44). DCs and NK cells share the expression of similar chemotactic receptors, such as CCR2, CCR4, CCR5, CXCR3, CCR7 and CMKLR1 and thus both cell types can potentially be recruited by common patterns of chemokines to inflammatory tissues and secondary lymphoid organs (13, 45). The relevance of this functional cooperation in immune response, including antiviral and antitumor immunity, is now well established (46–48). NK cells likewise modulate macrophage activation and polarization for example, by IFNγ production (49, 50). This regulatory role can also be effective on macrophage precursors. For example, NK cells can promote generation of Ly6C^high^ monocytes in several types of infections by regulating bone marrow precursors differentiation (51). Moreover, a subset of choline acetyltransferase (ChAT)^+^ NK cells was described to have a role in promoting CCR2^+^ Ly6C^high^ monocytes recruitment in central nervous system CNS during experimental autoimmune encephalomyelitis (EAE) and to reduce their disease-promoting effect by cell killing (52).

**Figure 1:**
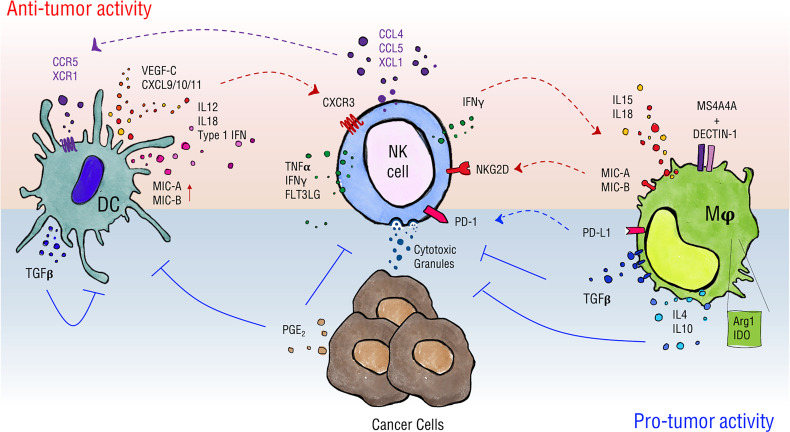
Schematics of NK cell interactions with DCs and macrophages. Myeloid cells can have different impacts on NK cell activity in the tumor context. DCs favor NK cell recruitment by release of CXCL9/10/11 and promote their activity in terms of proliferation, cytokine production and cytotoxicity through release of type 1 IFNs, IL-12 and IL-18, and by the expression of NKG2D ligands MIC-A/B. NK cells, in turn, can regulate DC infiltration by intratumor secretion of chemotactic factors such as CCL4, CCL5, XCL1 and FLT3LG and stimulate their maturation through TNFα and IFNγ secretion. In a similar way, macrophages release IL-15 and IL-18, and can express MIC-A/B when stimulated, thus supporting NK cell proper activation. Moreover, they can drive NK cell tumor cytotoxicity in a MS4A4A/Dectin-1-mediated recognition of cancer cells. On the other hand, DCs upregulate expression of TGF-β upon interaction with NK cells thus establishing negative feedback on activation of both cells involved. Macrophages shape NK cells phenotype contributing to the establishment of an immune-suppressive environment *via* the production of soluble factors such as TGF-β, IL-4, IL-10 and the expression of enzymes as Arg1 and IDO and of PD-L1, the ligand for PD-1 checkpoint receptor. Ultimately, cancer cells produce PGE_2_ that negatively affects DC and NK cell activity.

In analogy with adaptive immune cells, NK cells need priming to gain full activation potential. This action can be accomplished by IL-15 complexed to IL-15 receptor α-chain on the surface of cDCs and macrophages (52–55). Similarly, macrophage-derived IL-12 and IL-18 potently stimulate NK cell function and synergize for high level of IFNγ production (**Figure 1**) (56).

MDSCs represent myeloid cell subsets that strongly expand during tumor growth and that cooperates with the other tumor-associated myeloid cells to promote cancer development and the establishment of an immune-suppressive environment. MDSCs can affect NK cell phenotype in the tumor microenvironment and hijack their anti-tumoral potential by direct cell-to-cell interaction and/or by release of soluble factors such as PGE_2_, TGF-beta, IL-10, IL-6 and IL-23. The mechanisms underlying MDSC/NK functional interaction have been comprehensively reviewed recently (57, 58).

Due to space constraints, we will limit the description of NK cell crosstalk to DCs and macrophages in relation to the tumor context.

### NK Cell and DC Crosstalk in Cancer

Different DC subsets may have a different impact on NK cell activation, survival and proliferation mostly on the basis of the type of cytokine secreted. For instance, type I IFN produced by pDCs promotes NK cell cytotoxicity (59, 60), whereas IL-12 and IL-18 secreted by cDCs trigger cytokine production. cDC-derived IL-12, in synergism with IL-18, is required for IFNγ and NK cell cytotoxicity (61, 62).

NK-DC crosstalk may depend on both cell-to-cell contact and local release of cytokines (**Figure 1**). *In vitro*, NK cell activation requires the assembly of DC stimulatory synapses and local recruitment of IL-12/18-containing pre-assembled vesicles (63, 64). In addition, MIC-A and B expression by IFNα-stimulated DCs may drive NK cell activation (63). On the contrary, expression of ligands for the activating receptor NKG2D by DCs promotes NKG2D downmodulation and negatively impacts on optimal NK cell activation and tumor cytotoxicity in a melanoma mouse model (64).

The NK-DC cell ratio represents an important factor dictating either DC maturation or apoptosis. At high immature DC : NK ratio, DCs undergo maturation, whereas low ratio is associated with NK cell-mediated DC death (43, 65). In fact, immature DCs are susceptible to MHC class-I dependent NK lysis. On the other hand, mature DCs are resistant to autologous NK-cell killing through the upregulation of MHC class-I molecules during maturation. These observations suggested an immunoediting role of activated NK cells in the selection of immunostimulatory DCs (66). Upon maturation, DCs also upregulate the expression of adhesion molecules, such as CD155 and CD112 (the ligands of DNAM-1) (65), which play immune regulatory functions in tumor-infiltrating NK cells. In fact, tumor-infiltrating NK cells commonly express TIGIT, a checkpoint inhibitor receptor, that competes with DNAM-1 for the binding of CD155 and CD122 (48, 67–70).

**Figure 2:**
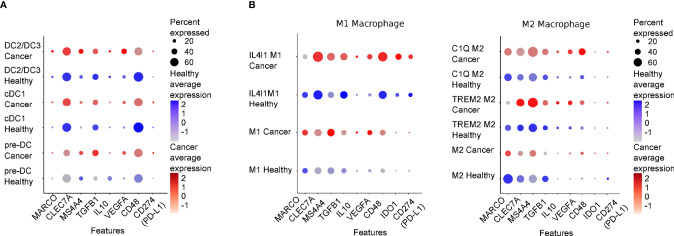
Expression levels of genes regulating DC/NK macrophage/NK interplay in healthy tissues and cancer lesions. DotPlot showing relative expression levels of genes presented in this review in Dendritic **(A)** and Macrophages **(B)** populations comparing a merge of scRNAseq of different healthy tissues (Healthy) or of several cancer types (Cancer: lung, colon, liver, breast, stomach and pancreas). The scRNA datasets presented in this review were derived from (38), and were downloaded from “https://gustaveroussy.github.io/FG-Lab/”. In detail, for the DC populations, classified in the MNP-VERSE dataset as pre-DC (progenitor DC), cDC1 and DC2/DC3 (that include cDC2 and DC3), while for the Macrophages populations (the function of which is described in the text), we relied on the “MoMac-VERSE” dataset. The indicated cell types were explored using Seurat (v4.04, https://satijalab.org/seurat/) within R version 4.0.5 (Shake and Throw). In blue and in red are shown the expression levels of healthy cancerous tissues, respectively. Color intensity is proportional to expression levels. MARCO, Macrophage receptor with collagenous structure; CLEC7A, C-type lectin domain family 7 member A or Dectin-1; IDO1, Indoleamine 2,3-dioxygenase.

Activated NK cells can in return stimulate DC maturation through the secretion of TNFα and IFNγ that increase the ability of DCs to produce cytokines and to prime Th1 and cytotoxic T lymphocyte (CTL) responses (43, 71, 72). In melanoma patients, NK cells regulate cDC1 survival and infiltration through the intratumoral production of FLT3LG; this action correlates with the efficacy of the therapeutic response to anti-PD1 immunotherapy (47). Furthermore, the interaction of DCs with IL-15-activated NK cells induces production of VEGF-C by DCs thus favoring lymphatic vessels formation *in vitro* (71). TGF-β is a highly pleiotropic cytokine whose role in physiology and pathological condition is strictly context-dependent; above all activities, TGF-β is a master regulator of the immune response and exerts a predominant inhibitory effect on a variety of immune cells. According to the analysis of the mononuclear phagocyte single cell RNA compendium from 41 datasets (MNP-VERSE), TGF-beta, CD48, Dectin-1 and MS4A4a are equally expressed by all DC subsets found in several human cancer types, i.e progenitor DC (pre-DC), cDC1 and CD11c^+^ cDC2 (that include cDC2 and DC3, DC2/DC3) [**Figure 2**, (38)]. Cytokines of the TGF-β family represent an important regulatory pathway of activated DCs (72); Activin A, a TGF-β family member, is one of the most upregulated cytokines during NK-DC interaction that was found to act as an important negative feedback mechanism for both NK cell and DC activation (69, 73). Interestingly, Activin A impaired NK cell-mediated killing of cancer cells *in vitro* and *in vivo* blockade of Activin A reduced lung metastases in a mouse model of lung metastasis (74).

### Role of Cell Migration in NK-DC Interplay in Cancer

Among DC subsets, cDC1 is the most effective in antitumor immunity (73) and the intratumoral abundance of cDC1 is associated with better patients’ survival (47, 73). The presence of cDC1s in the TME has been correlated with the presence of NK cells and with increased survival of melanoma, glioblastoma and neuroblastoma patients (75–78). Activated NK cells secrete several chemokines, such as CCL4, CCL5 and XCL1, that are responsible for the recruitment into the tumor of CCR5- and XCR1-expressing cDC1s (13, 45). Immunoreactive tumors are also characterized by the expression of CXCL9, CXCL10 and CXCL11 (45, 79, 80). Notably, these DC-derived chemokines recruit activated T and NK cells to promote efficient antitumor response (81, 82).

The potential relevance of the NK-cDC1 axis is emphasized by the fact that it can be part of tumor evasion strategies. For instance, it was shown in mouse models that tumor-derived PGE_2_ can decrease NK cell viability and chemokine production, and downregulate the expression of chemokine receptors by cDC1s, including *ccr5* and *xcr1* (48). This process reduces cDC1 recruitment and expansion *in situ* and alters their capacity to orchestrate an adaptive defensive response. Other immune evasion mechanisms include the DCs-mediated secretion of chemokines able to attract NK cell subsets that are less efficient in anti-tumor immunity. For instance, in lung and breast cancer, CXCL9 and CXCL10 preferentially recruit CD56^bright^, rather than the more cytotoxic CD56^dim^ NK cells (81). These data suggest that NK cell functional exhaustion observed within the TME may also depend on cell recruitment, but the effect of the biased migration of NK cell populations on cDC1 accumulation and survival is still not fully understood.

### Crosstalk Between NK Cells and Macrophages in Cancer

Macrophages are known to modulate NK cell function either through soluble factors or through cell-to-cell contact and, in turn, NK cell-derived inflammatory mediators can shape macrophage phenotype (83–86). Macrophages have been widely described as principal actors in directing NK cell activity also in the tumor context thus affecting tumor onset and growth and clinical outcomes (**Figure 1**) (87, 88). In regard of soluble factors, TAMs are described to express immune-suppressive cytokines such as IL-4, IL-10, TGF-β (89, 90) and mediators such as nitric oxide (87), to release a variety of chemokines (e.g. CCL2, CCL5, CCL18, CCL20) and to express enzymes whose activity is linked to an immune-suppressive environment as arginase1 and indoleamine-pyrrole 2,3-dioxygenase 1/2 (IDO 1/2) (88).

Among such factors, TGF-β produced by macrophages potently affects NK cell activity: indeed, *in vitro* co-culture of human NK cells with M2-polarized macrophages impaired secretion of IFNγ by CD56^bright^ NK cells through a mechanism mediated by soluble TGF-β (89). Monocyte/macrophage-derived TGF-β1 has also been described to induce functional impairment of NK cells infiltrating gastric cancer tumors that largely correlated with downmodulation of activating receptors such as CD16, NKp30, NKp46, NKG2D, DNAM-1, 2B4 and CD94 and reduced production of TNF-α and IFNγ (90).

In addition, macrophages and NK cells interplay in the tumor context can also be mediated by direct cell-to-cell contact. A study carried out on Hodgkin lymphoma (HL) and on diffuse large B-cell lymphoma (DLBCL) patients’ samples, described an immune evasion mechanism mediated by CD163^+^ monocytes/macrophages expressing PD-L1/PD-L2 that promoted NK cell phenotype switching towards an exhausted PD1^hi^CD3^-^CD56^hi^CD16^+/-^ phenotype evaluated in terms of expression of markers of activation. This was largely due to PD1 expression as assessed in TAMs and NK cells *in vitro* co-culture, since PD1 blockade was able to rescue NK cell activation, cytolysis and degranulation potential (91). This function can be related to macrophage distribution in the tumor lesion since PDL-1^+^ macrophages were found accumulated in clusters at the tumor invasive margin in lung adenocarcinoma, this correlating with impaired NK cell infiltration and features of reduced functionality (36). Furthermore, in human hepatocellular carcinoma, NK cell activity and lifespan were shown to be regulated by tumor-activated CD48^+^ monocytes/macrophages *via* the triggering of 2B4 that led to early NK cell activation and subsequent exhaustion. In support of such observation, *in vitro* treatment with anti-2B4 mAb at earlier time points attenuated NK cell activation and restored NK cell ability to secrete IFNγ and TNFα at later stages (92). Finally, macrophages can also express membrane-bound inhibitory cytokines: it has been recently demonstrated that metastasis-associated macrophages are able to suppress NK cell cytotoxicity through membrane-bound TGF-β in a mouse model of metastatic breast cancer (93).

Although TAMs have been widely associated with reduced NK cell activation, macrophages have also been shown to promote NK cell anti-tumor activity (56, 94, 95). For instance, a cell-to-cell contact-dependent mechanism involving DNAM1 and 2B4 and membrane-associated IL-18 expression was described to favor IFNγ secretion by NK cells. Bellora et al. studied TAM-NK cell cross-talk in ascites of ovarian cancer patients and reported that untreated TAMs induce low NK cell activation, while LPS-treated TAMs restored full NK cell activation. In addition, activated TAMs were shown to promote NK cell-dependent lysis of a NK cell-resistant ovarian cancer cell line (OVCAR-3), possibly through IL-12/IL-18-induced IFNγ production (96). In mouse tumor model, Chiba et al. revealed that N-glycans expressed on the surface of tumor cells initiate a signaling cascade on DCs and macrophages *via* the pattern recognition receptor Dectin-1 that ultimately leads to promotion of NK cell anti-tumoral activity (97). Interestingly, the tetraspan molecule MS4A4A, selectively expressed by M2-polarized macrophages was demonstrated to be required for the proper Dectin-1 mediated-NK cell activation in the metastatic foci thus sustaining the anti-tumor response (98). Considering the heterogeneity of macrophage subsets, populations associated with immunosuppression are likely to preferentially express molecules involved in NK cell inhibition. Indeed, analysis of the MoMac-VERSE in cancers shows that IL4I1 macrophages, a population of macrophages that co-express IDO1 and PD-L1, preferentially express TGF-b and CD48 as compared to C1Q^+^ and TREM^+^ macrophages which preferentially express MARCO and MS4A4a, respectively [**Figure 2** (38)].

Although it is clearly established that NK cells can regulate macrophage phenotype often leading to positive feedback loops (94), little is still known about NK cell activity in shaping macrophage function in the tumor context. A recent study revealed the ability of peripheral blood tumor-associated NK cells isolated from prostate cancer patients to release pro-inflammatory cytokines and chemokines that favor monocyte recruitment and M2-like polarization (95). In a glioma mouse model, NK cell activation by environmental stimuli promotes the polarization of CD11b^+^ myeloid cell subsets towards a pro-inflammatory phenotype through IFNγ secretion, thus enhancing the anti-tumor immune response (99).

### Role of Cell Migration in NK-Macrophage Interplay in Cancer

Tissue recruitment of monocytes or NK cells plays a critical role in their interplay and thus impacts on monocyte-derived inflammatory macrophage tissue accumulation and polarization and, reciprocally, NK cell activation in pathological conditions. For example, Kossmann and coworkers demonstrated that NK cells and monocytes play a key role in the vascular dysfunction induced by angiotensin II in a mouse model of hypertension. NK cells were attracted in the vascular wall by myelomonocytic cells and engaged with monocytes in inflammatory sites leading to mutual activation involving production of IFNγ (100). In addition, chemokines produced by CCR2^+^ Ly6C^hi^ monocytes and by a population of ChAT^+^ NK cells in the brain were proposed to lead to NK cell accumulation around the inflammatory monocytes with inhibition of their proinflammatory effect (56). Moreover, myeloid cells in lung tumors inhibit recruitment of NK cells upon chemotherapy by affecting vessel activation state through secretion of VEGF-A. In particular, VEGF-A reduced chemotherapy-induced recruitment of NK cells suppressing the release of chemerin, a NK cell chemoattractant, by lung endothelial cells in s.c. mouse tumor model (101). Also, a role for “patrolling” monocytes (PMo) in regulating NK cell anti-tumor activity has been delineated in a murine model of lung cancer where PMo induced NK cell recruitment through secretion of high levels of CCL3, CCL4, CCL5 and their activation in the tumor site. Furthermore, myeloid-specific deletion of Nr4a1, that is a master regulator for PMo development, showed reduced NK cell recruitment in tumor-bearing lungs and depletion of NK cells mitigated the differences in metastasis between wild-type and Nr4a1 knockout mice supporting the interplay between PMo and NK cells in tumor inhibition (102).

Thus, the repertoire of chemoattractant receptors on NK cells regulates their recruitment in diseased tissues and their local interaction with specialized population of monocytes.

## Conclusions and Therapeutic Perspectives

Tumor infiltration by NK and T cells is often associated with good prognosis. NK cells play an essential role in anti-tumor response, but exploitation of their anti-tumor activity needs full cell activation which is driven by elaborated environmental cues including interactions with other cells, such as DCs and macrophages (**Figure 1**). Understanding this complex cell-to-cell communication, that includes secretion of membrane-bound as well as soluble cytokines and chemokines, is essential to manipulate efficiently NK cell function in inflammatory or tumor clinical settings. Different types of cancers have developed molecular strategies to interfere with this network to avoid activation of anti-tumor immune cells, while favoring a local cell contexture able to sustain their growth. It is interesting that recognition of tumor determinants mediated by innate receptors can lead to complex outcomes in which NK cell activity can be affected both in a positive and in a negative manner. Furthermore, in this variegated scenario chemokine signals that drive TAM or DC accumulation are considered an appealing therapeutic target which is now very likely to affect NK cell function.

Restoring the functionality of NK-DC axis in the TME is a promising therapeutic approach to limit cancer progression (103). Both DCs and NK cells are crucial for the activation of effective anti-tumor immune response, but their protective functions are largely related to their ability to mutually interact and develop immune responses against cancer.

Intratumoral electroporation of a plasmid encoding IL-12 was effective in increasing the number of tumor infiltrating NK cells and cDC1s, boosting T cell response and improving anti-PD1 immunotherapy (104). Another therapeutic target under investigation is the XCR1-XCL1 axis, which can be exploited to increase the frequency of infiltrating cDC1s and consequently the numbers of intratumor T cells and NK cells (NCT00703222) (105).

Novel approaches to enhance the NK-DC axis involve the use of monoclonal antibodies (mAbs). The chimeric mAb cetuximab, targeting EGFR, activates NK cells and promotes DC maturation and CD8^+^ T cell priming, leading to tumor antigen spreading and Th1 cytokine release through a NK-DC crosstalk (106). Similarly, stimulation of CD137 delivers a robust costimulatory signal to both NK cells and DCs and improves cetuximab-mediated anti-tumor immune response and overall patients’ survival (107).

Targeting of macrophages in cancer has proven to be an interesting therapeutic approach to favor NK cell anti-tumor activity. A promising strategy has been to reduce TAMs in the tumor microenvironment to favor the anti-tumor activity of cytotoxic cells, for example by targeting CSF1R (colony stimulating factor 1 receptor) a receptor involved in TAMs tissue accumulation and survival or chemokine receptors involved in MDSCs tumor infiltration (108). Several phase 1 and 2 trials are currently under way either in monotherapy or in combination therapy (NCT01413022, NCT01804530, NCT01572519, NCT03557970, NCT02713529, NCT01444404, NCT02499328, NCT03689699).

A second encouraging approach is the reprogramming of TAMs into M1 macrophages to support immunotherapy effectiveness. For instance, poly I:C-treated macrophages were recently shown to express NKG2D ligands and to induce a highly NK cell-mediated cytotoxicity against tumor cells, but not against macrophages themselves (109). In *in vitro* and *in vivo* experiments on melanoma, M1 polarization promoted by nanovectors loaded with poly I:C correlated with increased tumor infiltration of activated NK and T cells and tumor regression (110). Finally, targeting of the scavenger receptor MARCO, expressed on a specific subpopulation of TAMs in a melanoma mouse model, was reported to affect the inflammatory phenotype and the metabolism of TAMs, resulting in increased serum levels of IL-15, activation of NK cells within the primary tumor and increased NK cell accumulation in metastatic lymph nodes (108). Further investigation on macrophage-NK cell crosstalk in the tumor microenvironment may furnish novel therapeutic strategies to unlock NK cell response against cancer cells or reverse TAMs pro-tumoral and immune-suppressive function.

## Author Contributions

ER, ML, LT, ADP, AS, SS, and GB wrote the manuscript. ER made **Figure 1**. ML made the analysis for **Figure 2**. All authors contributed to the article and approved the submitted version.

## Funding

This work was supported by the Italian Association for Cancer. Research (AIRC IG-20776 to SS and AIRC 5X1000 21147 to AS) by Italian Ministry for University and Research (Prin 20177J4E75 to SS and GB and 2017NTK4HY to ADP) and by Regione LAZIO Progetto Gruppi di Ricerca (n. 85-2017-15012 B81G18000840005).

## Conflict of Interest

The authors declare that the research was conducted in the absence of any commercial or financial relationships that could be construed as a potential conflict of interest.

## Publisher’s Note

All claims expressed in this article are solely those of the authors and do not necessarily represent those of their affiliated organizations, or those of the publisher, the editors and the reviewers. Any product that may be evaluated in this article, or claim that may be made by its manufacturer, is not guaranteed or endorsed by the publisher.
